# Psychotic Depression, Mannerisms and Alzheimer’s Disease: a case report

**DOI:** 10.1192/j.eurpsy.2024.1121

**Published:** 2024-08-27

**Authors:** A. Izquierdo De La Puente, P. del Sol Calderón, R. Fernandez Fernandez

**Affiliations:** ^1^Psychiatry, Hospital Universitario Puerta de Hierro de Majadahonda, Majadahonda; ^2^Psychiatry, Hospital Universitario Infanta Cristina, Parla, Spain

## Abstract

**Introduction:**

We present the case of a 56-year-old patient with two depressive episodes with psychotic symptomatology in a period of three years, who began with mania and developed Alzheimer’s disease.

**Objectives:**

The case is presented with the aim of providing a brief review of psychiatric symptomatology as a prodrome of Alzheimer’s disease.

**Methods:**

A 56-year-old patient, with no psychiatric antecedents of interest, who presented a depressive episode with psychotic symptoms, requiring admission to a short hospitalisation unit, as well as antidepressant treatment with sertraline at 200mg daily and olanzapine 20mg. He remained stable for two years and was able to withdraw treatment progressively. However, after remaining euthymic without pharmacological treatment for six months, he had another episode with psychotic symptoms. In this last episode, he did not require hospital admission, but he did require a change in antidepressant treatment, given that he did not tolerate treatment with sertraline. Treatment was therefore started with duloxetine 120mg, aripiprazole 20mg and as no clear improvement was observed, months later it was decided to use lamotrigine 100mg as a stabiliser.

**Results:**

In this last episode, despite the significant affective improvement and maintaining psychopathological stability, without presenting psychotic symptoms, the patient presented marked dysfunction in day-to-day life due to a striking attention deficit, lack of concentration and reduced short-term memory. At the same time, he also exhibits mannerisms which are observed in the consultation room, in the form of repetitive hand movements.

For these reasons, it was decided to request MRI and SPECT, obtaining results compatible with possible incipient cognitive deterioration.

**Conclusions:**

It seems that up to 40% of patients with dementia have depressive symptoms. It seems that depression at an advanced age may in fact be a prodromal symptom of dementia.

**Disclosure of Interest:**

None Declared

